# Non-degradative ubiquitination in Smad-dependent TGF-β signaling

**DOI:** 10.1186/2045-3701-1-43

**Published:** 2011-12-28

**Authors:** Liu-Ya Tang, Ying E Zhang

**Affiliations:** 1 Laboratory of Cellular and Molecular Biology, Center for Cancer Research, National Cancer Institute, Bethesda, Maryland 20892, USA

## Abstract

Transforming growth factor-β (TGF-β) signaling is tightly regulated at the level of post-translational modification to transmit quantitative difference in ligand concentration into proportional transcriptional output. Ubiquitination is one such modification with several E3 ubiquitin ligases implicated in TGF-β signaling in marking crucial pathway components for proteasomal degradation. However, ubiquitination, particularly in the mono- or oligo-ubiquitin modifying form, is also known to regulate incorporation of substrate proteins into signaling complexes that involved in DNA repair, kinase activation, and endocytosis. This review focuses on recent advances in understanding the role of such non-degradative ubiquitination in TGF-β signaling.

## Introduction

The transforming growth factor-β (TGF-β) superfamily consists of more than 30 secreted polypeptide growth factors including, but not limited to, TGF-βs, bone morphogenetic proteins (BMPs), activin/inhibin, growth and differentiation factors (GDFs) and nodal [1-3]. These cytokines regulate a wide array of biological processes ranging from cell proliferation and differentiation to migration and apoptosis [2,4]. Binding of ligands to the type II receptor (TβRII) recruits the type I receptor (TβRI), both of which are serine/threonine kinases, resulting in the formation of a heterotetrameric receptor complex. Within this complex, TβRI is activated by TβRII through phosphorylation, and the activated TβRI phosphorylates receptor-regulated Smad2 and Smad3 (R-Smads) at two serine residues in the C-terminus [2,5]. The C-terminal phosphorylation enables R-Smads to form heteromeric complexes with Smad4, which is commonly required for signaling by different R-Smads. The Smad complexes then accumulate in the nucleus to regulate target gene expression cooperatively with other transcription factors in a cell context-dependent manner [2,4,6]. A third class of Smads, mainly consisting of Smad6 and Smad7, negatively regulate TGF-β and BMP signaling by competing with R-Smads for binding to TβRI or targeting receptors to proteasomal degradation, therefore are named the inhibitory Smads (I-Smads) [7-10]. In addition to this canonical Smad-dependent signaling, TGF-β and its related ligands are also capable of activating small GTPases, mitogen-activated protein kinases, and phosphatidylinositol-3-kinase, initiating the so-called Smad-independent, noncanonical pathways [11].

Ubiquitin modification occurs in a three-step enzymatic reaction, which is catalyzed sequentially by ubiquitin activating enzyme (E1), ubiquitin conjugase (E2), and ubiquitin ligase (E3), resulting in attachment of ubiquitin to lysine residues of target proteins. Among these enzymes, the E3 ubiquitin ligase plays a crucial role in substrate recognition. Mammalian genomes contain more than 600 E3 ligases, which are divided into two categories based on sequence homology of the E2-binding domain: the Homologous to E6-AP Carboxyl Terminus (HECT) domain-containing E3s, and the Really Interesting New Gene (RING) finger domain-containing E3s [12]. Like phosphorylation, which can be reversed by the action of phosphatases, ubiquitination is also reversible by deubiquitination enzymes (DUBs) that cleave off ubiquitin moieties from substrates [13,14]. Different types of ubiquitin modifications are classified according to the number of ubiquitin moieties attached to substrates and the choice of lysine residue for the ubiquitin chain linkage. Poly-ubiquitination via the K48 linkage generally targets substrates for degradation by the 26S proteasome and regulates essentially all aspects of cellular functions[15], whereas mono- and oligo-ubiquitination or poly-ubiquitination via the K63 linkage mediate non-degradative events controlling DNA repair, kinase activation, and endocytosis [16]. Initially, various TGF-β pathway components were thought to be modified strictly by poly-ubiquitination and proteasome-mediated degradation that attenuates signaling output [17,18]; however, subsequent experiments demonstrate that some pathway components receive non-degradative ubiquitin modification in the form of mono-, oligo-, or even poly-ubiquitination that can lead to pathway activation under certain conditions[19,20]. The impact of degradative ubiquitination by different E3 ligases has been thoroughly summarized [17,18], so this review will focus on discussing recent advances in non-degradative ubiquitination in TGF-β signaling.

### Multiple mono-ubiquitination of Smad3 inhibits TGF-β-mediated transcriptional responses

**Figure 1:**
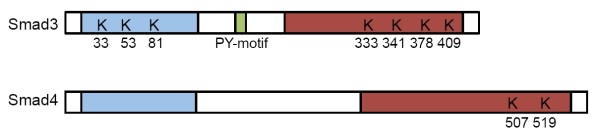
**Schematic diagram of ubiquitination sites in Smad3 and Smad4**. The conserved MH1 domain and MH2 domain are shown in blue and red, respectively. The non-conserved regions including the linker are shown in white. PY-motif, which is important for Smurf2 binding and ubiquitination, is shown in green.

**Figure 2:**
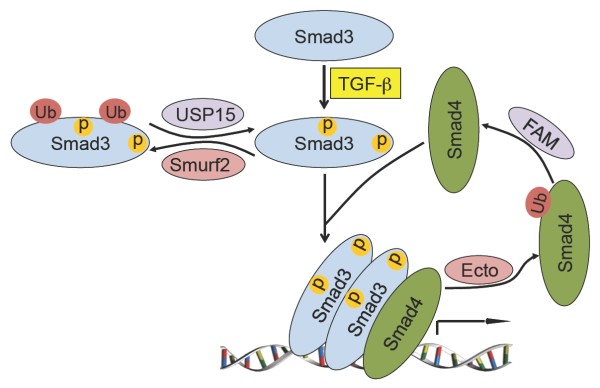
**A model for mono-ubiquitination in TGF-β signaling**. Upon TGF-β stimulation, Smad3 is phosphorylated at sites in both the linker and the C-terminal tail. Phosphorylation of T179 in the linker region potentiates Smurf2 binding and the subsequent mono-ubiquitination. Smad3 mono-ubiquitination can be reversed by USP15. On the other hand, mono-ubiquitination of Smad4 is induced by Ecto/Tif1γ, and reversed by FAM/USP9x. The unmodified Smad3 and Smad4 form a DNA binding complex that regulates target gene expression whereas mono-ubiquitinated Smad3 or Smad4 inhibits or disrupts the Smad complex formation.

Two recent studies have revealed that Smad3 undergoes multiple mono-ubiquitination, but this type of modification exerts no impact on Smad3 stability and phosphorylation. Instead, mono-ubiquitination of Smad3 modulates its transcriptional activity. Work from Stefano Piccolo's group identified multiple lysines of Smad3 including K33, K53, and K81 as recipients of mono-ubiquitin modification in HEK293T cells [21] (Figure 1). Ubiquitination at these residues affects DNA-binding activity of Smad3 since only unmodified Smad3 was pulled down by oligonucleotide probes containing the Smad-binding element [21]. Interestingly, mono-ubiquitination only inhibits direct interaction between Smad3 and DNA mediated by the Smad3 DNA-binding domain, but has no effect on indirect binding of Smad3 to DNA via other transcriptional factors. Formation of the Smad transcription complex favors Smad3 mono-ubiquitination, as this ubiquitination was shown to be enhanced by TGF-β but was inhibited in Smad4-depleted cells or in cells treated with the transcriptional inhibitors. On the other hand, ubiquitination of Smad3 causes it to be dissociated from DNA, because incubating Smad3 with either Smurf2 or NEDD4L, two previously characterized HECT E3 ligases in TGF-β signaling, released Smad3 in the ubiquitinated forms. Their work further showed that mono-ubiquitination of Smad3 can be reversed by de-ubiquitin enzyme, USP15, and knockdown of USP15 abolishes the recruitment of TGF-β-activated Smad complex on the chromatin (Figure 2). Thus, mono-ubiquitination can act in a self-limiting step during pathway activation and removal of ubiquitin modification by USP15 empowers Smad3 in the transcription response that it induces.

Concurrent to Piccolo's study, our group made an independent observation of Smad3 mono-ubiquitination in mouse embryonic fibroblasts (MEFs), and demonstrated that Smurf2 is the catalyzing enzyme[22]. Although both Smurf1 and Smurf2 were initially identified as E3 ligases that modulate TGF-β and BMP signaling, subsequent studies expanded the repertoire of their substrates to many other signaling pathways, and even in the process of epithelial to mesenchymal transition where TGF-β signaling is intimately involved, Smurf1 was found in a complex with Par6 to promote the localized degradation of RhoA at cell junction rather than acting through controlling the levels of Smads or TβRI [23]. These experimental observations raised the doubt on the physiological relevance of Smurfs in TGF-β/BMP signaling. Indeed, experiments with luciferase reporters monitoring transcriptional responses or Western blot analyzing Smad phosphorylation levels showed that loss of Smurf1 had little effect on TGF-β signaling [24], and data from Smurf1 and Smurf2 double knockout mice indicated that these two E3 ligases share a common function in regulating planar cell polarity by regulating asymmetric distribution of Prickl1 of the non-canonical Wnt pathway in cochlea and floor plate [25]. To clarify if indeed Smurf2 plays any role in TGF-β signaling and to address its physiological function, we generated Smurf2-deficient mice[22]. This allowed a rigorous loss-of-function examination of any possible role of this E3 ligase during embryogenesis and with biochemical analysis and reporter assays in isolated MEFs under *in vivo *conditions. By carefully examining the levels of various pathway components and transcriptional readouts in MEFs isolated from wild type, *Smurf2^-/-^*, and *Smurf1^-/- ^*mice, we found that Smurf2 is a specific E3 ligase responsible for multiple mono-ubiquitination of Smad3[22]. Interestingly, the level of ubiquitin modified Smad2 is not affected in *Smurf2^-/- ^*MEFs, even though Smad2 shares 92% sequence identity with Smad3, implying a high degree of substrate selectivity of Smurf2-mediated mono-ubiquitination reaction. Similar to the poly-ubiquitin modification of Smad3 induced by NEDD4L [26], interaction between Smurf2 and Smad3 as well as Smurf2-induced mono-ubiquitination of Smad3 requires phosphorylation of T179 and the adjacent PY motif (PPGY) in the linker region[22]. This requirement affords additional regulation of Smad3 activity to TGF-β, which has been shown to signal through cyclin-dependent kinases to control phosphorylation of T179 [27,28]. The sites of ubiquitination were mapped to four lysine residues in the MH2 domain, namely K333, K341, K378, and K409 [22](Figure 1). Since these lysines lie in the MH2 domain is positioned at the intermolecular interface of Smad3 complexes, it is conceivable that ubiquitination of these sites would inhibit formation of either the homomeric Smad3 or the heteromeric Smad3/Smad4 complexes [29]. This notion has been supported by evidence obtained with both *in vivo *co-immunoprecipitation experiments and *in vitro *GST pull-down assays[22]. Furthermore, we found that Smad3 accumulated more in the nucleus, and the rate of Smad3 export from the nucleus to the cytoplasm also decreased significantly in *Smurf2^-/- ^*MEFs upon TGF-β treatment, which offers a reasonable explanation for the elevated Smad3-dependent TGF-β transcriptional response that was observed in *Smurf2^-/- ^*MEFs[22]. Thus, upon TGF-β ligand binding to the receptors, phosphorylation of Smad3 at T179 in the linker region renders it a suitable substrate for Smurf2-mediated mono-ubiquitination (Figure 2). This modification interferes with Smad3 complex formation due to steric hindrance at the inter-molecular interface involving the MH2 domain, constituting a previously unnoticed negative feedback regulatory loop. These data unequivocally demonstrated a role of Smurf2 in modulating TGF-β signaling under in vivo conditions, albeit with a minimal role during embryogenesis. Further experimentation is required to identify the right environmental signals that invoke such fine tuning mechanism and, for that matter, its physiological significance.

### Itch mediated poly-ubiquitination enhances Smad2 phosphorylation by TβRI

Another HECT-domain E3 ligase, Itch, which shares structural similarity with Smurfs has been implicated in controlling TGF-β signaling. Loss of Itch leads to reduced responses to TGF-β signaling in fibroblasts, but in analogous to the situation in *Smurf2^-/- ^*cells, the levels of several Smads were found to be relatively constant in *Itch^-/- ^*MEFs comparing to those in *Itch^-/+ ^*control cells [19]. Itch can form a tripartite complex with Smad2 and the activated TβRI. Poly-ubiquitination of Smad2 by Itch in this complex was shown to enhance interaction between Smad2 and TβRI, thereby promoting phosphorylation of Smad2. Thus, unlike Smurfs, Itch exerts a positive influence on TGF-β signaling [19]. The significance of this work is that it demonstrates that Itch positively regulates TGF-β signaling by enhancing TβRI induced-Smad2 phosphorylation in an ubiquitination-dependent manner. However, the underlying mechanism that controls interaction between Itch and Smad2, and how ubiquitination facilitates Smad2-TβRI interaction are not clear.

### Poly- and oligo-ubiquitin modifications of Smad4 differentially regulate its activity

Smad4 was initially identified as a frequent deletion target in pancreatic carcinomas, and Smad4 deletions or mutations were also found in colon, breast, and lung cancers, albeit with less frequency [30]. Consistent with these findings, studies of mice carrying various targeted mutations indicated that loss of Smad4 results in tumor formation in multiple organs and tissues[31-33]. In cancer cells, a panel of 4 missense mutations in the MH1 domain of Smad4 identified in human pancreatic and colorectal cancers was reported to render the mutant Smad4 a better substrate for poly-ubiquitin modification and proteasome-mediated degradation [20]. However, in normal cells wild type Smad4 is actually a quite stable protein, as it is a poor substrate of poly-ubiquitination and turns-over very slowly even in the presence of cycloheximide [20]. Instead, wild type Smad4 is subjected to mono- and oligo-ubiquitin modification in the MH2 domain. However, the literature differs significantly with regard to the impact of mono- and oligo-ubiquitin modification on Smad4 function with one report from Moustakas' group suggesting that it enhances formation and transcriptional activity of the Smad3-Smad4 complex [20] whereas another from Piccolo's concluding in quite an opposite direction that it disrupts the functional Smad3-Smad4 complex [34]. Crystallographic and calorimetric data indicate that the transcriptionally active Smad complexes are heteromeric trimers comprising one Smad4 and two phosphorylated R-Smads. The interface between these Smad moieties is formed between the C-terminal tail and the L3-loop of the MH2 domain [29]. The ubiquitin attachment site of Smad4 ubiquitination in Moustakas' study was determined to be K507 by mass spectrometry [20]. However, mutational studies by Piccolo's group showed that the most important recipient lysine for mono-ubiquitination is the neighboring K519 [34] (Figure 1). Given the close proximity of either of these two lysines to the actual contacting residues in the MH2 domain, it is difficult to conceive that a bulky mono-ubiquitin chain could enhance the heteromeric bounding within the Smad complexes; nevertheless, Itch poly-ubiquitination of Smad2 has been shown to promote the interaction between Smad2 and TβRI as discussed above [19]. Notwithstanding the functional discrepancy between these two studies, one thing is clear that Smad4 can be modified either by poly-ubiquitin chains or by mono-ubiquitin. These two forms of modification have differential impact on Smad4 function.

The potential influence of mono-ubiquitin modification on Smad4 function suggests that it is likely regulated to afford additional control of TGF-β signaling. Indeed, in a siRNA-based nonbiased screen for novel TGF-β signaling modulators among 75 known or predicted DUBs, FAM/USP9x was identified, and an antagonizing E3 ligase, Ectodermin (Ecto)/Tif1γ was also identified through candidate gene approach [34] (Figure 2). Comparative experiments in Drosophila, zebrafish, and mammalian cells showed that this pair of counter-acting E3 and DUB are functionally conserved, underscoring the evolutionary importance of Smad4 mono-ubiquitin modification.

### Concluding remark and perspective

In contrast to an abrupt "turning-off" role of the degradative ubiquitination, the non-degradative ubiquitination exerts versatile regulation of TGF-β signaling through various mechanisms. It can be a positive regulation by promoting the receptor-mediated phosphorylation of Smad2 or a negative regulation by disrupting Smad complex formation. Despite the remarkable recent progress in this highly active area of research, many questions remain. For example, what is the temporal order for ubiquitins to modify Smad proteins in response to TGF-β, and what is structural basis for poly-ubiquitin chains to enhance Smad complex formation? It is also unclear what makes Smad2 such a poor substrate for Smurf2-mediated ubiquitination, although sharing a high degree of sequence identity with Smad3 and is capable of binding to Smurf2? Finally, what determines the specificity of Itch and Smurf2, the two related HECT-domain E3 ligases, in selecting different substrates to regulate TGF-β signaling? Rigorously addressing these questions will be challenging, but the final outcome will undoubtly provide further insight into how TGF-β signaling is regulated.

The poly- and oligo-ubiquitin chains discussed in this review are all form via the K48 linkage. Other internal lysine residues, such as K63, could also be utilized to support the addition of homogenic or heterogenic chain linkage for the ubiquitin modification of various pathway components, raising the possibility that TGF-β signaling can be regulated by yet other forms of ubiquitin modification. High-resolution systematic mass spectrometry analysis coupled with efficient ubiquitin affinity purification will prove to be instrumental in this emerging area of research.

## Competing interests

The authors declare that they have no competing interests.

## Authors' contributions

LYT and YEZ wrote the manuscript. Both authors read and approved the final manuscript.
